# Wnt/β-catenin signaling suppresses expressions of *Scx*, *Mkx*, and *Tnmd* in tendon-derived cells

**DOI:** 10.1371/journal.pone.0182051

**Published:** 2017-07-27

**Authors:** Yasuzumi Kishimoto, Bisei Ohkawara, Tadahiro Sakai, Mikako Ito, Akio Masuda, Naoki Ishiguro, Chisa Shukunami, Denitsa Docheva, Kinji Ohno

**Affiliations:** 1 Division of Neurogenetics, Center for Neurological Diseases and Cancer, Nagoya University Graduate School of Medicine, Nagoya, Japan; 2 Department of Orthopaedic Surgery, Nagoya University Graduate School of Medicine, Nagoya, Japan; 3 Department of Molecular Biology and Biochemistry, Division of Dental Sciences, Graduate School of Biomedical and Health Sciences, Hiroshima University, Hiroshima, Japan; 4 Experimental Trauma Surgery, Department of Trauma Surgery, University Regensburg Medical Centre, Regensburg, Germany; National Cancer Center, JAPAN

## Abstract

After tendon injuries, biomechanical properties of the injured tendon are not fully recovered in most cases. Modulation of signaling pathways, which are involved in tendon development and tendon repair, is one of attractive modalities to facilitate proper regeneration of the injured tendon. The roles of TGF-β signaling in tendon homeostasis and tendon development have been elucidated. In contrast, the roles of Wnt/β-catenin signaling in tendon remain mostly elusive. We found that the number of β-catenin-positive cells was increased at the injured site, suggesting involvement of Wnt/β-catenin signaling in tendon healing. Activation of Wnt/β-catenin signaling suppressed expressions of tenogenic genes of *Scx*, *Mkx*, and *Tnmd* in rat tendon-derived cells (TDCs) isolated from the Achilles tendons of 6-week old rats. Additionally, activation of Wnt/β-catenin reduced the amounts of Smad2 and Smad3, which are intracellular mediators for TGF-β signaling, and antagonized upregulation of *Scx* induced by TGF-β signaling in TDCs. We found that Wnt/β-catenin decreased *Mkx* and *Tnmd* expressions without suppressing *Scx* expression in *Scx*-programmed tendon progenitors. Our studies suggest that Wnt/β-catenin signaling is a repressor for tenogenic gene expressions.

## Introduction

Tendon injuries, due to degeneration with aging or overuse, are frequently observed in clinical settings and remain a challenge in orthopedic trauma [1]. Even after long-term observations, the structure and strength of repaired tendon do not show full recovery, and the patient rarely regains a pre-injury range of motion [2]. Nowadays, cell-based tissue engineering is one of attractive strategies for the musculoskeletal regeneration. In tendon engineering, mesenchymal stem cells (MSCs) and tendon-derived cells are suitable modalities for this purpose. Recently, human MSCs or genetically modulated MSCs, are investigated for cell implantation to assist tendon repair in a rat model of tendon injury [1]. In addition, elucidation of the signaling pathways involved in normal tendon development may lead to identification of extracellular factors, which can be potentially applied to develop a therapeutic strategy to rejuvenate the biomechanical properties of degenerated tendon [3, 4]. However, relatively little is known about the mechanisms directing tendon development and extracellular factors controlling gene expressions of tendon cells.

Mature adult tendons are normally characterized by low cellular density. Approximately 90–95% of cells in human tendon are comprised of tendon-specific cells, which are referred to tendon cells or tenocytes. The cells are derived from MSCs, which are terminally differentiated and responsible for synthesis and turnover of tendon fibers comprised of collagens fibril and glycoproteins [4]. Scleraxis (encoded by *Scx*), Mohawk (*Mkx*), and Tenomodulin (*Tnmd*) are expressed through the lineage differentiation during development, and are required for the maturation of collagen fibrils. Scleraxis, a bHLH transcription factor, is highly expressed in tendon progenitors throughout differentiation [5]. Loss of *Scx* results in severe disruption of force-transmitting tendons with less collagen fibers, as well as defective maturation of the enthesis [6, 7]. Mohawk, a homeobox protein, plays a critical role in tendon differentiation by regulating type I collagen production in tendon cells. *Mkx*^-/-^ mice show hypoplastic tendon tissues with down-regulation of type I collagen expression and small collagen fibril diameters [8]. Tenomdulin is a type II transmembrane glycoprotein containing a cleavable C-terminal cysteine-rich anti-angiogenic domain [9, 10] and is predominantly expressed in tendon and ligament tissues [11]. Loss of *Tnmd* in tendon results in enlarged calibers of collagen fibrils, suggesting impaired maturation of collagen fibrils [12, 13].

At the repairing site of injured tendon in dog, extracellular growth factors including transforming growth factor-beta (TGF-β), epithelial growth factor (EGF), platelet-derived growth factor (PDGF), insulin growth factor (IGF), basic fibroblast growth factor-2 (FGF2), and vascular endothelial growth factor (VEGF) are detected by immunohistochemical analysis [14]. These growth factors are also detected in injured tendons at the mRNA and protein levels in chick [15] and at the mRNA level in rabbit [16]. During tendon healing, these growth factors enhance synthesis of collagens and proteoglycans, as well as proliferation and/or differentiation of tendon cells [17]. In a rat rotator cuff healing model, FGF-2 stimulates the growth of tenogenic cell population that gives rise to *Tnmd*^+^ cells [18]. TGF-β (TGF-β1, β2 and β3 ligands bind to a heteromeric receptor comprised of TGFβR1 and TGFβR2, which phosphorylates and activates the transcription factors, Smad2 or Smad3, in TGF-β1 signaling. All three isoforms can enhance the production of collagens in equine tendon-derived cells [19]. Intracellular TGF-β signaling is up-regulated in limb tendon cells during development [20]. Deficiency of Smad3 in *Smad3*^-/-^ mice shows disruption of normal tendon architecture and results in reduced expressions of *Scx* and *Mkx* in tendon tissue [3].

Wnt ligands are a large family of secreted glycoproteins, which regulate differentiations of MSCs in embryonic development and crucial aspects of cell differentiation during adult tissue homeostasis [21]. Wnt ligands activate the canonical Wnt/β-catenin signaling, as well as additional non-canonical pathways [22, 23]. In the absence of Wnt lingands,β-catenin is steadily phosphorylated by casein kinase 1 (CK1) and glycogen synthase kinase 3 (GSK3) in a degradation complex assembled by Axin1 and adenomatous polyposis coli (APC), and is subsequently degraded through the ubiquitin/proteasome pathway [24]. The Wnt ligands or (2'Z,3'E)-6-bromo-indirubin-3'-oxime (BIO), a GSK3 inhibitor, suppresses phosphorylation of β-catenin, and subsequently suppresses degradation of β-catenin. Consequently, β-catenin is accumulated in the cytoplasm and then translocated into the nucleus to interact with T-cell factor/lymphoid enhancing factor (TCF/LEF) and activate transcription of Wnt/β-catenin-target genes. *Wls*, a responsible gene for secretion of Wnt ligands, is essential for distal tendon induction in limb buds in mouse embryos [25]. However, little is known about the roles of Wnt/β-catenin signaling in tendon cells in adult mouse.

We here show that accumulation of β-catenin protein is observed in tendon cells adjacent to the injured site. In primary cells isolated from adult rat tendon, activation of Wnt/β-catenin signaling reduces gene expressions of *Scx*, *Mkx*, and *Tnmd*. Wnt/β-catenin also reduces total and phosphorylated Smad2/3 proteins, and antagonizes TGF-β1-induced *Scx* expression in the primary cells. We propose that activation of Wnt/β-catenin signaling attenuates differentiation of tendon cells by suppressing gene expressions of *Scx*, *Mkx*, and *Tnmd*.

## Materials and methods

### Tendon-injury model and immune-detection of β-catenin

**Fig 1:**
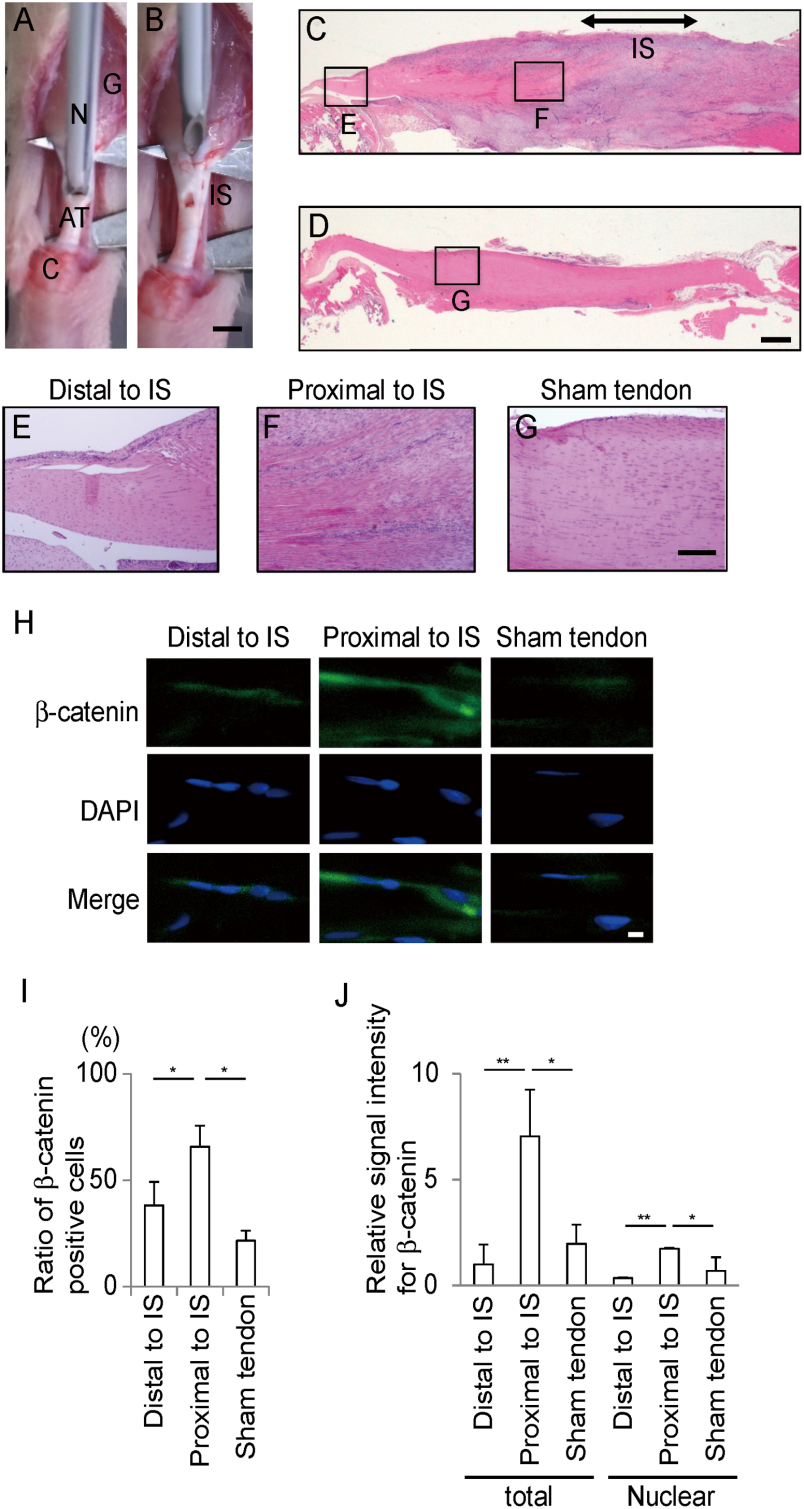
Staining for β-catenin proteins is increased adjacent to the injured site in the rat Achilles tendon. **(A, B)** The Achilles tendon (AT) between calcaneum (C) and gastrocnemius (G) was punctuated with a 14-gauge needle (N) at the injured site (IS). Scale bar = 2 mm. **(C, D)** Hematoxylin-eosin staining of sagittal sections of injured **(C)** and sham-operated tendons **(D)** on postoperative day 14. Position of IS with abundant inflammatory cells is indicated by a double-headed arrow in **C**. Tendons are placed with the distal side on the left and the proximal side on the right. Scale bar = 200 μm. **(E-G)** High magnification of the areas indicated in **C** and **D**. **(E)** An image distant from IS and close to the calcaneum. **(F**) An image adjacent to IS and near the center of the tendon. **(G)** An image in sham-operated tendon. Scale bar = 200 μm. **(H)** Immunostaining for β-catenin (green) with DAPI (blue). Scale bar = 5 μm. **(I)** The ratio of β-catenin-positive cells in the field of ~36,000 μm^2^ is indicated by mean and SD (*n* = 3 rats each). The numbers of cells counted in the ~36,000 μm^2^-image field for Distal to IS, Proximal to IS, and Sham tendon are 21–56 cells, 29–72 cells, and 28–55 cells, respectively. The number of β-catenin positive cells is divided by the number of DAPI-positive cells in the field. **(J)** Mean and SD (*n* = 3 rats each) of intensities of total cellular and nuclear β-catenin signals of the tendon cells indicated in **(I)**. Each intensity is normalized by the number of DAPI-positive cells. *p* < 0.05 by one-way ANOVA. *p < 0.05, **p < 0.01 by Tukey-Kramer post-hoc test.

All animal studies were approved by the Animal Care and Use Committee of the Nagoya University. Sprague Dawley (SD) rats (6-week-old, male, weighting 200–230 g, Japan SLC, Inc.) were anesthetized with 2.5% sevoflurane. Under sterile conditions, the right Achilles tendon was punctured at the midpoint between calcaneus and gastrocnemius muscle by a 14-gauge needle and the skin was sutured with 6–0 nylon thread (injured tendon) (Fig 1A and 1B). The left Achilles tendon remained uninjured, but the skin and synovium over the tendon were incised (sham-operated tendon). On postoperative day 14, rats were euthanized with controlled flow-rate carbon dioxide, and the Achilles tendons were isolated and stained with hematoxylin-eosin. Serial sections were incubated with a rabbit antibody against β-catenin (BD Transduction Laboratories, 1:200 dilution) at 4°C overnight and then incubated with a secondary donkey antibody against rabbit IgG (H+L) conjugated with Alexa Fluor 488 (Thermo Fisher #A21206, 1: 1000 dilution) at room temperature for 1 hr. The sections were mounted in VectaShield containing 2 ng/ml diamidino-2-phenylindole (DAPI, Vector Laboratories, Peter-borough, UK) and visualized using the IX71 (Olympus) microscope.

Signals for β-catenin were quantified in three rats for each group using the MetaMorph software (Molecular Device). Each rat was analyzed by three blinded observers. We analyzed two areas (distal and proximal to the injured site) of the injured tendon, and the middle area of the sham-operated tendon. Each area was comprised of ~36,000 μm^2^. Signals less than 4 μm in diameter were ignored as non-specific signals, and signals more than 4 μm in diameter were taken as positive signals. DAPI-staining was used to localize the nucleus and to count the number of cells. When the intensity of β-catenin in the nucleus was similar to or more than that in the cytoplasm, the cell was counted as a nuclear β-catenin-positive cell. The number of nuclear β-catenin positive cells in a ~36,000 μm^2^ -area was normalized by the number of DAPI-positive cells in the same area.

### Primary culture of tendon-derived cells (TDCs)

SD rats (6-week-old males weighting 200–230 g) were euthanized with controlled flow-rate carbon dioxide, and TDCs were isolated from the Achilles tendons, as reported elsewhere [26, 27]. Briefly, after removing peritendineum, tendon tissue was cut into ~1-mm pieces and placed in a 10-cm culture plate with Dulbecco’s Modified Eagle’s Medium (DMEM, Life Technologies) supplemented with 10% fetal bovine serum (FBS) and Pen Strep (Life Technologies). The plates were placed in a humidified incubator with 5% CO_2_ at 37°C. After 14 days, the cells were detached with trypsin-EDTA for 5 min, and seeded in a 15-cm plate. After the cells were similarly passaged two times, the cells were seeded in a six-well plate at a density of 2 × 10^5^ cells/well for each experiment.

### Culture of human *SCX*-programmed tendon progenitors (hMSC-Scx cells)

MSCs isolated from human bone marrow cells were immortalized by retrovirally transducing human *TERT* gene (hMSC) [28], and were lentivirally transduced with FLAG-*SCX* cDNA to make hMSC-Scx cells ([29]). Similarly, FLAG cDNA was transduced into hMSC to make hMSC-Mock cells. Tendon-related collagens and proteoglycans were sufficiently expressed in hMSC-Scx cells compared to hMSC-Mock cells [29]. hMSC-Scx cells were seeded in a six-well plate at 2 x 10^5^ cells/well with Minimum Essential Medium, Alpha Modification + GlutaMAX (MEM α, GlutaMAX, no Nucleosides, Life Technologies) supplemented with 10% FBS and Pen Strep (Life Technologies).

### Treatment of cultured cells with chemical compounds and recombinant proteins

TDCs and hMSC-Scx cells were cultured in the medium stated above, and were supplemented with 0.5–4 μM BIO (Sigma, #B1686), 50 ng/ml human recombinant Wnt3a protein (R&D Systems, #5036-WN), 5–20 μM IWR1-endo (IWR, Tocris #3532), 0.5–8 ng/ml human recombinant TGF-β1 protein (R&D Systems, #100-21C), and/or 0.5–8 μM SD208 (Wako, 193–16331).

### Total RNA extraction and quantitative RT-PCR analysis

TDCs and hMSC-Scx cells were treated with chemical compounds and recombinant proteins for 72 hrs and 48 hrs, respectively. Total RNA was isolated using QuickGene-800 (Kurabo). The first strand cDNA was synthesized with ReverTra Ace (Toyobo). We quantified rat or human mRNAs for *Scx*, *Mkx*, and *Tnmd* as tenogenic genes, and for *Axin2* as an indicator of activated Wnt/β-catenin signaling using LightCycler 480 (Roche) and SYBR Green (Takara). The mRNA levels were normalized by rat *Gapdh* or human *GAPDH*. Primer sequences for rat *Axin2* [30], *Scx*, *Runx2*, *Vegf* [2], *Mkx*, *Tnmd*, and *Gapdh* [31], as well as human *AXIN2*, *MKX*, *TNMD* [32], and *GAPDH* [29] are shown in S1 Table.

### Western blotting

TDCs and hMSC-Scx cells were treated with BIO for 48 hrs. TGF-β1 (0 or 2 ng/ml) was added 30 min before harvesting cells. The cells were lysed with the ice-cold RIPA Lysis Buffer (Santa Cruz) with phosphatase inhibitors (PhosSTOP, Roche) as previously reported [33]. Whole cell lysates were separated on SDS-PAGE and transferred to a nitrocellulose membrane followed by immunoblotting with antibodies against Phospho-Smad2/Smad3 (p-Smad2/3) (Cell Signaling Technology, #8828, 1:1000 dilution), Smad2/3 (Cell Signaling Technology, #5678, 1:1000 dilution), and β-actin (Santa Cruz, sc-47778, 1:1000 dilution). Band intensities were quantified in three independent experiments for each group with ImageQunat LAS4000 Mini (GE Healthcare Life Sciences), and were normalized by β-actin or total-Smad2/3.

### Statistical analysis

Two groups were compared by unpaired Student’s *t*-test. Multiple groups were analyzed by one-way analysis of variance (ANOVA) followed by Tukey-Kramer post-hoc test. *P*-values less than 0.05 were considered statistically significant. All statistical analyses were performed with SPSS Statistics 23 (IBM).

## Results

### β-catenin is accumulated in tendon cells adjacent to the injured site of the Achilles tendon

To examine the activity of Wnt/β-catenin signaling in tendon cells after tendon injury, we analyzed accumulation of β-catenin in a rat model of tendon injury. The Achilles tendon of rat was punctuated by a needle on day 0 (Fig 1A and 1B), and sagittal sections were stained with hematoxylin-eosin and immunostained for β-catenin on day 14. Hematoxylin-eosin staining revealed that inflammatory cells infiltrated tendon fibers at the edge of the injured site as previously reported (Fig 1C) [3], but not in sham-operated tendon (Fig 1D). Tendon fibers located distant from the injured site (Fig 1E), as well as of sham-operated tendon (Fig 1G), remained well organized, while tendon fibers adjacent to the injured site were disorganized (Fig 1F). Immunostaining for β-catenin revealed that β-catenin was accumulated in tendon cells adjacent to the injured site, while the signals were significantly weak in tendon cells distant from the injured site and in sham-operated tendon (Fig 1H–1J). These results suggest that Wnt/β-catenin signaling is activated in healing tendon cells.

### Wnt/β-catenin signaling suppresses gene expressions of *Scx*, *Mkx*, and *Tnmd* in TDCs

**Fig 2:**
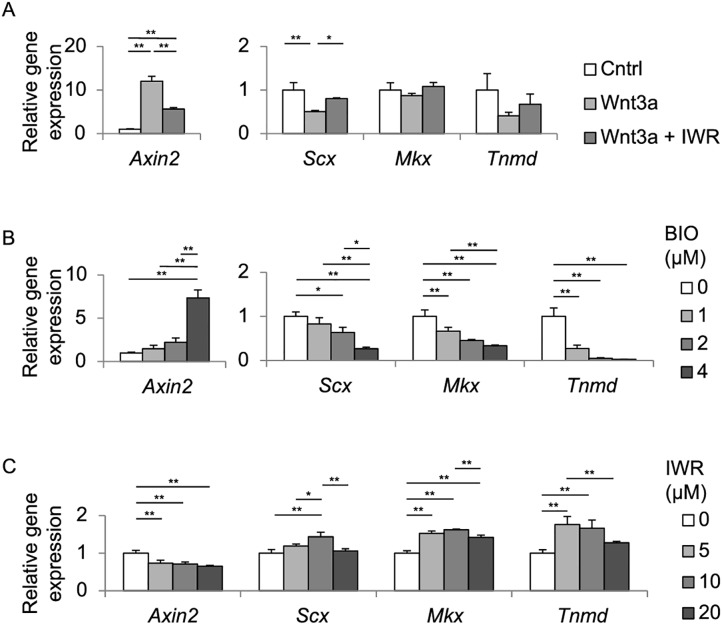
Activation of Wnt/β-catenin signaling decreases mRNA expressions of *Scx*, *Mkx* and *Tnmd* in rat TDCs. Relative expressions of *Axin2*, *Scx*, *Mkx*, and *Tnmd* in TDCs treated with 50 ng/ml Wnt3a with or without 5 μM IWR (an inhibitor of β-catenin) **(A)**, 0 to 4 μM BIO (an activator of β-catenin) **(B)**, or 0 to 20 μM IWR **(C)** for 72 hrs. **(B)** Increasing concentrations (1, 2, and, 4 μM) of BIO increased *Axin2* expression to 150%, 220%, and 730% of that without BIO, respectively. **(C)** Increasing concentrations (5, 10, and, 20 μM) of IWR decreased *Axin2* expression to 73%, 71%, and 66% of that without IWR, respectively. As BIO was dissolved in DMSO, all samples in **B** were incubated under 0.008% DMSO. Each mRNA expression is normalized by *Gapdh* mRNA. Mean and SD are indicated (*n* = 3 wells each). Tukey-Kramer post-hoc test (*p < 0.05, **p < 0.01) is indicated only when *p* < 0.05 by one-way ANOVA.

To evaluate the effects of Wnt/β-catenin signaling in tendon cells, TDCs were isolated from the Achilles tendon of 6-week-old male SD rats, and were treated with Wnt3a, BIO, and/or IWR. BIO is an inhibitor for kinase activity of GSK3α/β, and activates Wnt/β-catenin signaling. IWR stabilizes the β-catenin degradation complex, and inhibits Wnt/β-catenin signaling. We first confirmed that Wnt3a and increasing concentrations of BIO induce *Axin2* expression to activate Wnt/β-catenin signaling (Fig 2A and 2B). Conversely, suppression of Wnt/β-catenin signaling by IWR was corroborated by suppression of Wnt3a-induced *Axin2* expression (Fig 2A) and of endogenous *Axin2* expression (Fig 2C). In contrast to *Axin2*, expression of *Scx* was significantly suppressed and expressions of *Mkx* and *Tnmd* trended to be suppressed by Wnt3a (Fig 2A). Similarly, the suppression was partially rescued by IWR. We next added variable concentrations of BIO or IWR without adding exogenous Wnt3a in TDCs. BIO up-regulated and IWR down-regulated expression of *Axin2* in a dose-dependent manner (Fig 2B and 2C). In contrast to *Axin2*, BIO down-regulated expressions of *Scx*, *Mkx*, and *Tnmd* in a dose-dependent manner (Fig 2B). Contrarily, IWR up-regulated expressions of *Scx*, *Mkx*, and *Tnmd*, but dose-dependence was observed up to 10 μM IWR (Fig 2C). Thus, Wnt/β-catenin signaling suppresses expressions of tenogenic *Scx*, *Mkx*, and *Tnmd* genes in TDCs.

A previous study shows that Wnt/β-catenin signaling is implicated in tendon ossification [34]. We thus analyzed the effect of Wnt3a on expressions of osteogenic genes, *Runx2* and *Vegf* [2]. In contrast to *Scx*, *Mkx*, and *Tnmd*, however, BIO had no significant effect on expressions of *Runx2* and *Vegf* in TDCs in 3 days (S1 Fig).

### TGF-β signaling induces expression of *Scx* in TDCs

**Fig 3:**
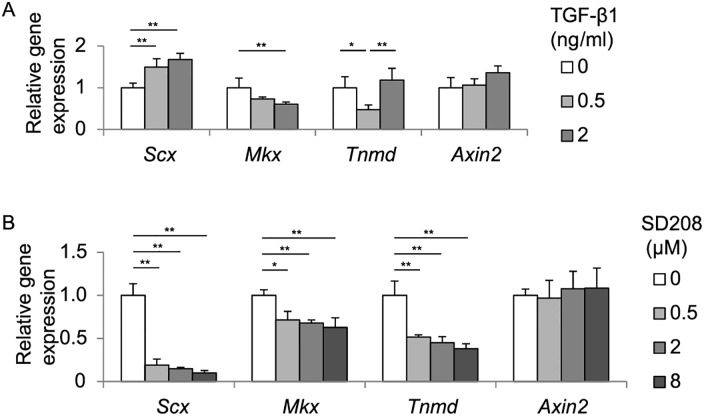
Activation of TGF-β signaling increases mRNA expression of *Scx*. Relative expressions of *Scx*, *Mkx*, *Tnmd*, and *Axin2* in TDCs treated with 0 to 8 ng/ml TGF-β1 **(A)** or 0 to 8 μM SD208 (an inhibitor of TGF-β signaling) **(B)** for 72 hrs. Each mRNA expression is normalized by *Gapdh* mRNA. Mean and SD are indicated (*n* = 3 wells each). Tukey-Kramer post-hoc test (*p < 0.05, **p < 0.01) is indicated only when *p* < 0.05 by one-way ANOVA.

TGF-β signaling is a regulator for tenogenic gene expressions in tendon cells during development [35, 36]. TGF-β signaling is a potent inducer of *Scx* both in limb tendons in mouse embryos, as well as in C3H10T1/2 cells [35, 36]. To test the effects of TGF-βsignaling on *Scx*, *Mkx*, and *Tnmd* in rat TDCs, TDCs were treated with mouse recombinant TGF-β1, a ligand for TGF-β signaling, and SD208, a chemical inhibitor against receptors for TGF-β ligands, for 72 hrs. TGF-β1 increased and SD208 decreased *Scx* expression in a dose-dependent manner (Fig 3A and 3B). In contrast, TGF-β1 and SD208 had no effect on *Axin2* expression. Thus, activation of TGF-β signaling induces *Scx* expression in TDCs, which is independent of Wnt/β-catenin signaling. We also found that TGF-β1 and SD208 both decreased expression of *Mkx* in a dose-dependent manner. In addition, TGF-β1 and SD208 had variable and suppressive effects on *Tnmd* expression, respectively, in TDCs. As TGF-β1 and SD208 have the opposing effects on TGF-β signaling, the observed changes in expressions of *Mkx* and *Tnmd* cannot be simply accounted for by modulation of TGF-β signaling. Similarly, as neither TGF-β1 nor SD208 affected *Axin2* expression (Fig 3A and 3B), which is a marker gene for activated Wnt/β-catenin signaling, Wnt/β-catenin signaling is unlikely to be involved in modulated expressions of *Mkx* and *Tnmd*.

### Wnt/β-catenin signaling antagonizes activation of TGF-β signaling and partially cancels the TGF-β-mediated induction of *Scx* expression in TDCs

**Fig 4:**
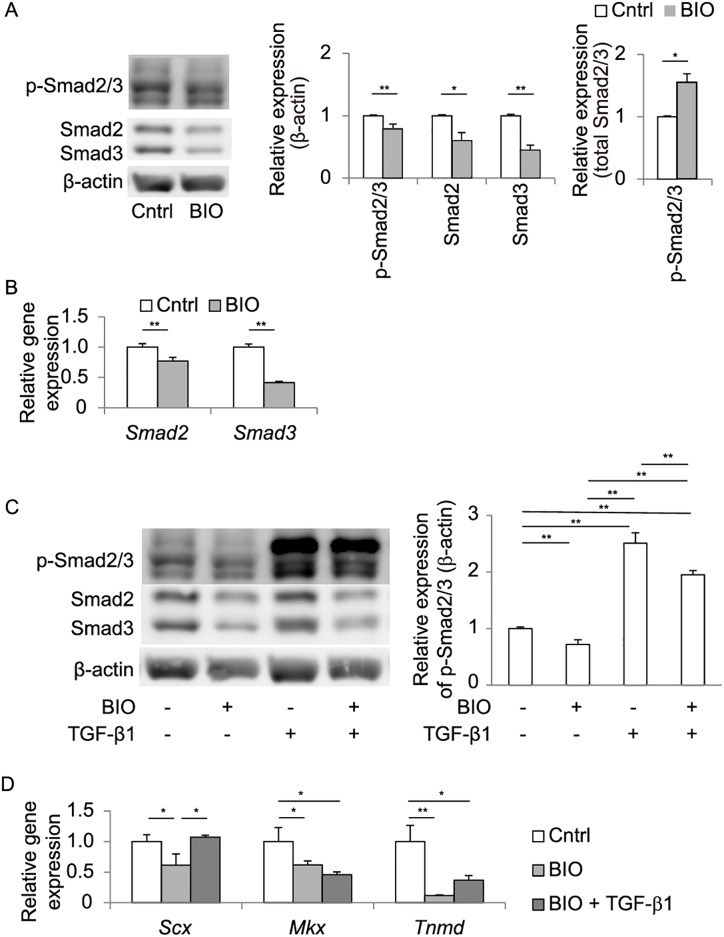
Activation of Wnt/β-catenin signaling reduces total and phosphorylated Smad2/3 proteins, and suppresses *Scx* expression in TDCs. **(A, C)** Western blotting for Smad2, Smad3, and phosphorylated Smad2/3 (p-Smad2/3) proteins. Rat TDCs were treated with 4 μM BIO for 48 hrs **(A)** or with 4 μM BIO for 48 hrs followed by treatment with 2 ng/ml TGF-β1 for 30 min **(C)**. The mean and SD of band intensities of three independent wells are indicted. Band intensities are normalized by β-actin or total-Smad2/3. **(B)** Quantitative RT-PCR of *Smad2* and *Smad3* in TDCs treated with 4 μM BIO for 24 hrs. Mean and SD are indicated (*n* = 3 wells each). **(D)** Quantitative RT-PCR of *Scx*, *Mkx*, and *Tnmd* in TDCs treated with 4 μM BIO with or without 2 ng/ml TGF-β1 for 48 hrs. Each mRNA expression is normalized by *Gapdh* mRNA. Mean and SD are indicated (*n* = 3 wells each). All samples in **A-D** were added with 0.008% DMSO, because BIO was dissolved in DMSO. **(A, B)** *p < 0.05, ** *p* < 0.01 by unpaired Student’s *t*-test. **(C, D)** *p* < 0.05 by one-way ANOVA for all groups. *p < 0.05, **p < 0.01 by Tukey-Kramer post-hoc test.

Since Wnt/β-catenin signaling and TGF-β signaling down- and up-regulated *Scx* expression, respectively (Figs 2 and 3), we investigated a relationship between the two signaling pathways in TDCs. First, we examined expressions of Smad2 and Smad3, which are intracellular mediators of TGF-β1 signaling [37]. Western blotting showed that BIO treatment for 48 hrs decreased Smad2, Smad3, and phosphorylated Smad2/3 proteins (Fig 4A). The ratio of phosphorylated-to-total Smad2/3, however, was increased by BIO (Fig 4A). As BIO decreased gene expressions of *Smad2* and *Smad3* (Fig 4B), down-regulation of phosphorylated Smad2/3 was likely due to down-regulation of gene expressions of Smad2/3 and not to reduced phosphorylation of Smad2/3. Even after 48-hr treatment with BIO, TGF-β1 was able to induce phosphorylation of Smad2/3 (lanes 2 and 4 in Fig 4C). Similarly, 48-hr treatment with both TGF-β1 and BIO rescued BIO-induced suppression of *Scx* expression (Fig 4D). Wnt/β-catenin signaling is thus likely to antagonize activation of TGF-β signaling, and to partially cancel the TGF-β-mediated induction of *Scx* expression in TDCs.

We also examined the effect of TGF-β signaling on Wnt3a-mediated suppression of *Mkx* and *Tnmd* in TDCs. We observed that TGF-β1 had no significant effect on Wnt3a-mediated suppression of *Mkx* and *Tnmd* (Fig 4D).

### Wnt/β-catenin signaling down-regulates *MKX* expression in hMSC-Scx cells

hMSC-Scx cells were previously generated by ectopic expression of human *SCX* cDNA into immortalized human MSCs [1]. hMSC-Scx cells are able to constitute advanced cellular organization and matrix maturation in the injured rat Achilles tendon. To investigate the effects of Wnt/β-catenin signaling and TGF-β1 signaling on tenogenic gene expressions in hMSC-Scx cells, we treated hMSC-Scx cells with activators and/or inhibitors used for TDCs (Figs 2 and 3). As previously reported [29], the exogenous *SCX* (FLAG-*SCX*) and endogenous *TNMD* were over-expressed in hMSC-Scx cells compared to hMSC-Mock cells, which expressed only FLAG cDNA (Panel A in S2 Fig). On the other hand, *MKX* expression was minimally increased in hMSC-Scx cells (Panel A in S2 Fig).

**Fig 5:**
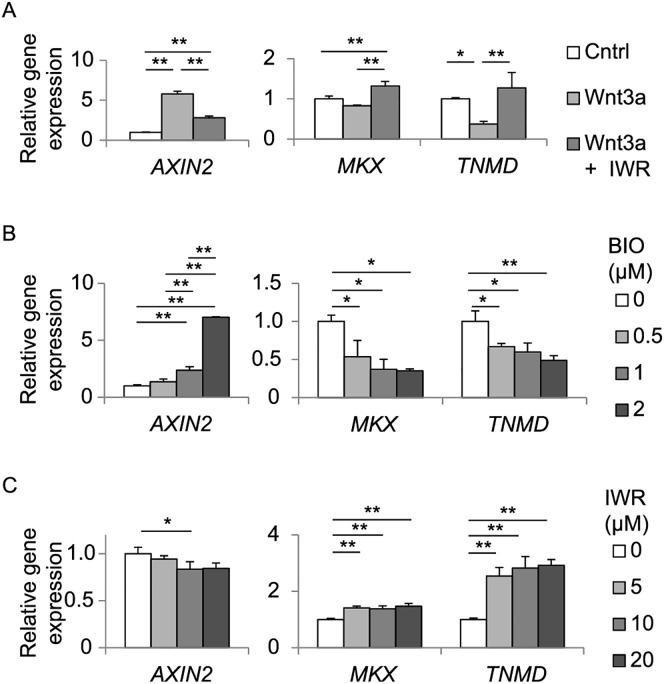
Wnt/β-catenin reduces *MKX* and *TNMD* expressions in *SCX*-programmed tendon progenitors (hMSC-Scx cells). hMSC-Scx cells were treated with either Wnt3a, BIO (an activator of β-catenin), IWR (an inhibitor of β-catenin), or their combination, as in Fig 2. Relative expressions of *AXIN2*, *MKX*, and *TNMD* in hMSC-Scx cells treated with 50 ng/ml Wnt3a with or without 5 μM IWR **(A)**, 0 to 2 μM BIO **(B)**, or 0 to 20 μM IWR **(C)** are indicated. As BIO was dissolved in DMSO, all samples in **B** were incubated under 0.004% DMSO. **(B)** Increasing concentrations (0.5, 1, and 2 μM) of BIO increased *AXIN2* expression to 130%, 240%, and 700% of that without BIO, respectively. **(C)** Increasing concentrations (5, 10, and 20 μM) of IWR decreased *AXIN2* expression to 94%, 84%, and 84% of that without IWR, respectively. Each mRNA expression is normalized by *GAPDH* mRNA. Mean and SD are indicated (*n* = 3 wells each). *p* < 0.05 by one-way ANOVA for all groups. *p < 0.05, **p < 0.01 by Tukey-Kramer post-hoc test.

We first confirmed that Wnt3a and BIO up-regulated and IWR down-regulated *AXIN2* expression in hMSC-Scx cells (Fig 5A–5C). We also corroborated that expressions of exogenous FLAG-*SCX* and total (exogenous plus endogenous) *SCX* were not affected by BIO (Panel B in S2 Fig). Wnt3a suppressed expressions of *MKX* and *TNMD*, and IWR cancelled the suppression in hMSC-Scx cells (Fig 5A), as we observed in TDCs (Fig 2A). Consistently, BIO reduced and IWR increased expressions of *MKX* and *TNMD* in a dose-dependent manner (Fig 5B and 5C). Wnt/β-catenin signaling was thus able to suppress expressions of *MKX* and *TNMD* in *SCX*-overexpressing hMSC-Scx cells.

**Fig 6:**
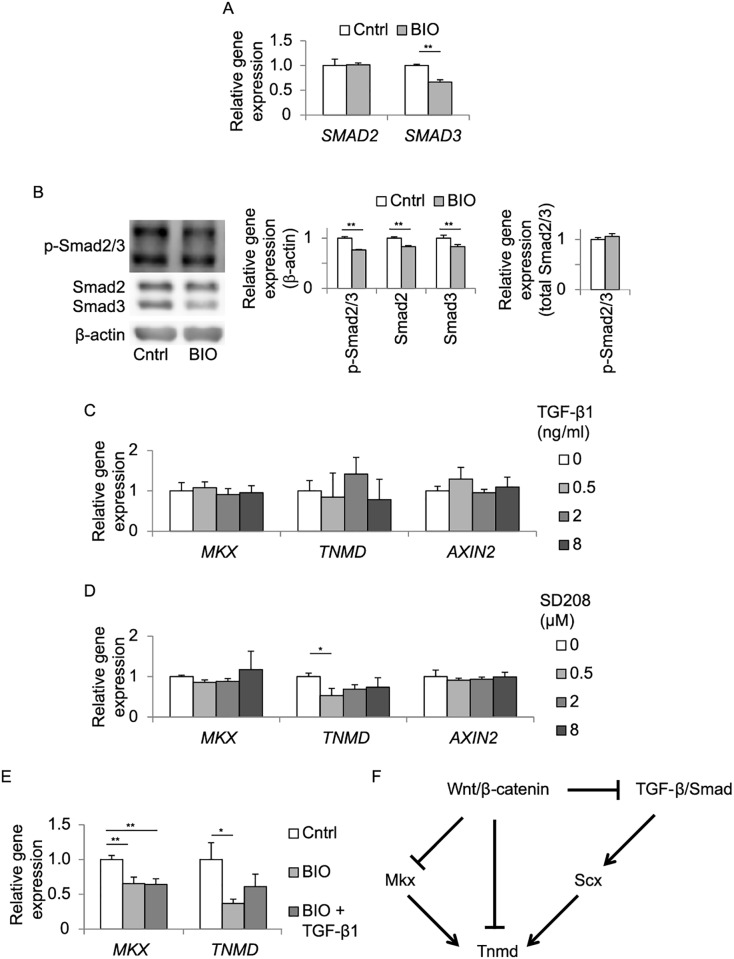
TGF-β signaling has no significant effect on *MKX* and *TNMD* expressions in *SCX*-programmed tendon progenitors (hMSC-Scx cells). **(A)** Quantitative RT-PCR of *SMAD2* and *SMAD3* expressions in hMSC-Scx cells treated with 1 μM BIO for 24 hrs. Each mRNA expression is normalized by *GAPDH* mRNA. **(B)** Western blotting for Smad2, Smad3, and phosphorylated Smad2/3 (p-Smad2/3) in hMSC-Scx cells treated with 1 μM BIO for 48 hrs, as in Fig 4A and 4B. Band intensities are normalized by β-actin or total-Smad2/3. **(C, D)** Relative expressions of *AXIN2*, *MKX*, and *TNMD* in hMSC-Scx cells treated with 0 to 8 ng/ml TGF-β1 **(C)**, or 0 to 8 μM SD208 (an inhibitor of TGF-β signaling) **(D)**, for 48 hrs, as in Fig 3. **(E)** Quantitative RT-PCR of *MKX* and *TNMD* expressions in hMSC-Scx cells treated with 1 μM BIO with or without 2 ng/ml TGF-β1 for 48 hrs. Each mRNA expression is normalized by *GAPDH* mRNA. All samples in **A**, **B**, and **E** were incubated under 0.002% DMSO. **(A, B)** **p < 0.01 by unpaired Student’s *t*-test. **(C-E)** Tukey-Kramer post-hoc test (*p < 0.05, **p < 0.01) is performed only when *p* < 0.05 by one-way ANOVA. **(A-E)** Mean and SD of three independent wells are indicated. **(F)** A schematic showing induction and suppression of gene expressions of *Scx*, *Mkx*, and *Tnmd* by TGF-β signaling and Wnt/β-catenin in tendon cells. Wnt/β-catenin suppresses expression of *Tnmd* via *Scx* and *Mkx*. Direct suppression of *Tnmd* by Wnt/β-catenin remain to be determined. Arrows from *Scx* and *Mkx* to *Tnmd* are based on a previous report observed in mouse bone marrow-derived mesenchymal stem cells (BMMSCs) [38].

We also examined a relationship between Wnt/β-catenin signaling and TGF-β signaling in hMSC-Scx cells. We first corroborated that, in hMSC-Scx, BIO suppressed mRNA levels of *SMAD2/3* (Fig 6A), which subsequently suppressed protein levels of total and phosphorylated Smad2/3 (Fig 6B), as we observed in TDCs (Fig 4A and 4B). We next confirmed that TGF-β1 induced phosphorylation of Smad2/3 in hMSC-Scx cells (lanes 1 and 3 in Panel C in S2 Fig), as we observed in TDCs (lanes 1 and 3 in Fig 4C). TGF-β1 and SD208, however, had no effects on expressions of *MKX*, *TNMD*, and *AXIN2* (Fig 6C and 6D). Similarly, TGF-β1 did not cancel the BIO-induced suppression of *MKX* and *TNMD* expressions (Fig 6E). Taken together, Wnt/β-catenin signaling suppresses *MKX* and *TNMD* expressions without modulating TGF-β signaling in hMSC-Scx cells.

## Discussion

During 7–21 days after injury of the patellar tendon in mice, expressions of tenogenic genes of *Scx*, *Mkx*, and *Tnmd*, as well as genes for fibril assembly are reduced in the injured tendon [7]. This report prompted us to investigate which growth factors regulate gene expressions in tendon cells after tendon injury *in vivo*. Injection of collagenase in the rat tendon induces intense expressions of Wnt3a and β-catenin proteins on postoperative days 14–28 [34]. Here we applied a mechanical damage to the rat Achilles tendons, and observed abnormal derangement of tendon fibers and infiltration of inflammatory cells on postoperative day 14. We also observed abnormal accumulation of β-catenin in tendon cells adjacent to, but not distant from, the injured site (Fig 1). Wnt/β-catenin signaling is thus activated by tendon injury.

We indicated putative roles of Wnt/β-catenin signaling in tendon cells in Fig 6F, which are deduced from our current study and a previous report [38]. Exogenous Wnt3a activates the *Scx* promoter through β-catenin in C3H10T1/2 cells, which are pluripotent mouse embryonic fibroblasts [25]. BIO, an activator for Wnt/β-catenin signaling, induces *Tnmd* expression, but not *Scx* or *Mkx* expression, in equine bone marrow-derived MSCs cultured in collagen gels [21]. We found that Wnt/β-catenin signaling suppressed expressions of *Scx*, *Mkx*, and *Tnmd* in rat TDCs (Fig 2), and *Mkx* and *Tnmd* in hMSC-Scx cells (Fig 5). A previous report shows that Wnt3a treatment for 10 days increases alkaline phosphatase activity, calcium nodule formation, and expressions of osteogenic markers including osteocalcin (*Bglap*) and alkaline phosphatase (*Alpl*) in TDCs [34]. We found that BIO treatment for 3 days (72 hrs) had no effect on expressions of *Runx2* and *Vegf*, which are early markers for osteogenic differentiation in TDCs (S1 Fig). Thus, Wnt/β-catenin signaling is likely to suppress tenogenic differentiation and maturation in ~3 days, which subsequently leads to osteogenic differentiation of TDCs in ~10 days. Implantations of *Scx*-expressing or *Mkx*-expressing MSCs facilitate regeneration of the injured tendon with large collagen fibrils [39, 40]. As Wnt/β-catenin signaling is likely to be deleterious for injured tendon, therapeutic suppression of Wnt/β-catenin signaling, if possible, and the subsequent enhancement of *Scx*, *Mkx*, and *Tnmd* expressions is expected to facilitate tendon healing.

Each isoform of TGF-β ligands stimulates gene expressions of *Scx*, *Mkx* and *Tnmd*, and is recognized as an inducer of tendon differentiation [41, 42]. In mouse embryonic limbs, TGF-β2 and -β3, as well as their receptors, are expressed in and around the region where tendons are developed, and TGF-βsignaling is required for *Scx* expression in tendon progenitors [35]. In mouse TDCs cultured in collagen gels, TGF-β1 increases *Scx* and *Mkx* expressions in a dose-dependent manner [41, 42]. In human TDCs, TGF-β1 increases *Scx* expression, but not *Mkx* or *Tnmd* expression [36, 42], whereas TGF-β3 increases both *Scx* and *Tnmd* expressions [43]. In mouse C3H10T1/2 cells, TGF-β2 increases *Scx* expression [44]. In our study, TGF-β1 increased *Scx* expression in rat TDCs (Fig 3), but did not regulate *Mkx* and *Tnmd* expressions in hMSC-Scx cells (Fig 6A and 6B). To summarize, TGF-β signaling increases *Scx* expression in all four cell types (mouse, human, and rat TDCs, and mouse C3H10T1/2 cells), as well as in mouse embryonic limbs. In contrast, TGF-β signaling increases *Mkx* in mouse TDCs and *Tnmd* in human TDCs, whereas TGF-β signaling has no effect in other cell types. The effect of Wnt/β-catenin signaling on TGF-β signaling has not been examined in tendon cells or tendon tissues, but has been reported in two other MSC-derived cells. In chondrocytes, overexpression of β-catenin significantly inhibits TGF-β signaling [45]. In myofibroblasts, Wnt3a induces differentiation by up-regulating TGF-β signaling in a β-catenin-dependent manner [46]. We found that activation of Wnt/β-catenin signaling by BIO decreased the amounts of Smad2 and Smad3 in rat TDCs and hMSC-Scx cells (Fig 4AB). Wnt/β-catenin signaling and TGF-β signaling the opposite effects on *Scx* expression in rat TDCs (Fig 4C). These results suggest that Wnt/β-catenin signaling antagonizes TGF-β signaling to regulate *Scx* expressions in TDCs (Fig 6F). In contrast, Wnt/β-catenin signaling suppresses *MKX* and *TNMD* expressions independently of TGF-β signaling in MSC-Scx cells (Fig 6A–6E).

We showed that Wnt/β-catenin signaling reduced *MKX* and *TNMD* expressions even in SCX-overexpressing hMSC-Scx cells (Fig 5B). Thus, Wnt/β-catenin signaling-mediated downregulation of *MKX* and *TNMD* is independent of SCX. We also showed that Wnt/β-catenin signaling decreased phosphorylated Smad2/3 (Fig 4A) by suppressing their mRNA levels (Fig 4B). As TGF-β1 increased phosphorylated Smad2/3 (Fig 4C), as well as *Scx* in TDCs (Fig 3A), Wnt/β-catenin signaling is likely to suppress *Scx* by inhibiting TGF-β/Smad signaling. A previous report shows that, in mouse bone marrow-derived mesenchymal stem cells (BMMSCs), overexpression of *Scx* increases *Tnmd*, but not *Mkx* [38]. Conversely, overexpression of *Mkx* in BMMSCs increases *Tnmd*, but not *Scx* [38]. Thus, *Scx* has no effect on *Mkx* and *vice versa* in BMMSCs. Our current study and the previously report [38] point to the notion that *Scx* and *Mkx* independently induce expression of *Tnmd* (Fig 6F). We propose that identification of a small compound that suppresses Wnt/β-catenin signaling is expected to lead to development of a novel therapeutic option to facilitate regeneration of injured tendons.

## Supporting information

S1 Fig Activation of Wnt/β-catenin signaling has no significant effect on expressions of osteogenic genes in TDCs. Quantitative RT-PCR analysis for *Runx2 and Vegf* in TDCs treated with 0 to 4 μM BIO. Each mRNA expression is normalized by *Gapdh* mRNA. Mean and SD are indicated (*n* = 3 wells each). No significant difference is observed by one-way ANOVA.
(EPS)

S2 Fig Wnt/β-catenin and TGF-β signaling show no significant effects on expressions of *total SCX* and *FLAG-SCX* transgene in *SCX*-programmed tendon progenitors (hMSC-Scx cells). **(A)** Quantitative RT-PCR for FLAG-tagged *SCX* transgene (*F-SCX*), *total SCX* (a sum of the endogenous gene and the transgene), *MKX*, and *TNMD* in hMSC-Scx cells compared to control MSCs (hMSC-Mock cells). *p < 0.05, **p < 0.01 by unpaired Student’s *t*-test. **(B)** Quantitative RT-PCR analysis for *F-SCX* and *total SCX* in hMSC-Scx cells treated with 0 to 2 μM BIO or 0 to 8 ng/ml TGF-β1 for 48 hrs. Each mRNA expression is normalized by *GAPDH* mRNA. **(C)** Western blots for Smad2, Smad3, and phosphorylated Smad2/3 (p-Smad2/3). hMSC-Scx cells were treated with 1 μM BIO for 48 hrs followed by treatment with or without 2 ng/ml TGF-β1 for 30 min. Band intensities are normalized by β-actin. **(B, C)** Tukey-Kramer post-hoc test (*p < 0.05, **p < 0.01) is indicated only when *p* < 0.05 by one-way ANOVA. **(A, B, C)** Mean and SD are indicated (*n* = 3 wells each).
(EPS)

S1 Table Primer sequences. (DOCX)
