# Supplementary material for: Wnt/β-catenin signaling suppresses expressions of Scx, Mkx, and Tnmd in tendon-derived cells
Source: PLoS One. 2017 Jul 27;12(7):e0182051. doi: 10.1371/journal.pone.0182051 (PMC5531628; doi:10.1371/journal.pone.0182051)
Supplement: S1 Table — (DOCX) [file pone.0182051.s003.docx]

|  | Gene | Forward primer | Reverse primer |
| --- | --- | --- | --- |
| Rat | *Axin2* | CTGGCTATGTCTTTGCACCA | AGGAGGGATTCCATCTACGC |
|  | *Scx* | TCATCCCGACCGAGCCAGCA | CCGCAGGCTTCACCCACCAG |
|  | *Tnmd* | TGGAAATGGCACCGATGAAAC | GCAGGAACCCAAATCACTGACTG |
|  | *Mkx* | TTTACAAGCACCGTGACAACCC | ACAGTGTTCTTCAGCCGTCGTC |
|  | *Gapdh* | GGGTGTGAACCACGAGAAAT | ACTGTGGTCATGAGCCCTTC |
|  | *Runx2* | CCGCACGACAACCGCACCAT | CGCTCCGGCCCACAAATCTC |
|  | *Vegf* | TTCAGAGCGGAGAAAGCATT | GAGGAGGCTCCTTCCTGC |
|  |  |  |  |
| Human | *AXIN2* | TACCGGAGGATGCTGAAGGC | CCACTGGCCGATTCTTCCTT |
|  | *FLAG-SCX* | ACTACAAGGACGACGATGAC | CCGTGTTCACGCTGTTGGTG |
|  | *SCX* | AACACGGCCTTCACTGCGCTG | CAGTAGCACGTTGCCCAGGTG |
|  | *TNMD* | ATTCAGAAGCGGAAATGGCACTGA | TAGGCTTTTCTGCTGGGACCCAA |
|  | *MKX* | TTTACAAGCACCGTGACAACCC | GCTAAGCCGTTCAGCATTGC |
|  | *GAPDH* | CAACTACATGGTTTACATGTTC | GCCAGTGGACTCCACGAC |
